# Neural correlates of reward processing distinguish healthy youth at familial risk for bipolar disorder from youth at familial risk for major depressive disorder

**DOI:** 10.1038/s41398-022-01800-9

**Published:** 2022-01-24

**Authors:** Akua F. Nimarko, Aaron J. Gorelik, Kayla E. Carta, Mark G. Gorelik, Manpreet K. Singh

**Affiliations:** 1 Stanford University School of Medicine, Stanford, CA United States; 2 University of Florida, Gainesville, FL United States

**Keywords:** Bipolar disorder, Depression, Neuroscience

## Abstract

Youth at familial risk for bipolar disorder (BD-risk) and major depressive disorder (MDD-risk) have aberrant reward processing, a core feature of these mood disorders. Whether BD risk differentiates from MDD risk in reward processing merits further study. We compared neural activation and connectivity during anticipation and outcome of monetary gain and loss during fMRI using the Monetary Incentive Delay (MID) Task among BD-risk (*n* = 40), MDD-risk (*n* = 41), and healthy comparison youth (HC) (*n* = 45), in the absence of any lifetime or current history of psychopathology [mean age 13.09 ± 2.58, 56.3% female]. Participants completed the MID task at baseline and were followed for behavioral and clinical outcomes over 4.37 ± 2.29 years. Region-of-interest (ROI) analyses conducted using anatomically defined thalamus, ventrolateral prefrontal cortex, nucleus accumbens, and putamen seeds showed that relative to MDD-risk and HC, BD-risk had decreased activation of the thalamus during anticipation of monetary gain [*F*(2,118) = 4.64, *p* = 0.01 (FDR-corrected *p* = 0.04)]. Psychophysiological interaction analyses revealed that BD-risk had less connectivity between the thalamus and left middle frontal gyrus (*Z* > 3.1, *p* < 0.001) and left-superior temporal gyrus (*Z* > 3.1, *p* < 0.05) compared with MDD-risk. Voxelwise, BD-risk had decreased activation in the cerebellum during anticipation and outcome of monetary gain relative to MDD-risk and HC (*Z* > 3.1, *p* < 0.001; *Z* > 3.1, *p* < 0.01). In BD-risk, decreased thalamic connectivity was associated with increased impulsivity at baseline and reduced prosocial behavior at follow-up. Reduced thalamic activation and connectivity during reward processing may distinguish familial risk for BD from familial risk for MDD and represent early markers of vulnerability that may herald social dysfunction later in adolescence.

## Introduction

Bipolar disorder (BD) and major depressive disorder (MDD) are serious and persistent conditions that when developed during childhood and adolescence [1, 2] result in worse outcomes compared with adult onset [3, 4]. Reward-processing dysfunction is a core feature of BD and MDD [5, 6]. Whereas anhedonia is a transdiagnostic symptom manifestation of reward dysfunction that may be observed in MDD and BD [7, 8], hedonism is a distinct feature of BD [9, 10]. Further, although initial depressive presentations of these disorders may overlap, familial aggregation patterns [11] and nonoverlapping symptoms suggest distinct disruptions in reward processes. Unfortunately, transdiagnostic reward deficits that point to common illness features can lead to delays in accurate diagnosis and appropriate treatment. A missed BD diagnosis treated with antidepressants could lead to adverse side effects such as a switch to mania in BD and BD-risk individuals [12, 13]. Elucidating reliable, early reward-processing deficits distinguishing BD from MDD would provide insights for unique predisposing factors for symptom trajectories and lead to more refined approaches to accurate diagnosis and treatment selection.

Limbic, paralimbic, striatal, and cortical regions (nucleus accumbens (NAcc), putamen, thalamus, and ventrolateral prefrontal cortex (VLPFC)) have been implicated in reward-network dysfunction in BD and MDD [5, 14–17]. Family history is a known risk factor for developing a major mood disorder [18–22] and youth at risk for BD may be exposed to chaotic family environments that may impact frontostriatal networks [23]. For these reasons, imaging studies of offspring of parents with BD (BD-risk) or MDD (MDD-risk) are ideally suited to investigate early and distinct neural endophenotypes of reward processing that precede symptom onset [24, 25]. Among youth with BD and BD risk, aberrant functional activations and connectivities in the thalamus, pregenual cingulate cortex, and frontal regions during reward processing have already been reported compared with healthy and psychiatric comparison groups [16, 25, 26]. These studies and others suggest that hyper- and hyposensitivity to reward observed during mood episodes [27] and aberrant prediction-error signaling [28] may be related to aberrant function in the subcomponents of neural reward circuits known to regulate these behaviors. Although there has been much focus on the regulation of striatal activity and connectivity by the prefrontal cortex, the thalamus may play a unique role in distinguishing unipolar from bipolar depression [29]. For example, we found that youth shortly after their first manic episode showed thalamic hypoactivity during reward processing compared with healthy adolescents [16]. This potentially unique pattern of thalamic dysfunction has also been reported in individuals with BD when compared with individuals with MDD [29, 30]. Youth with or at-risk for MDD rather show blunted striatum, insula, and increased dorsal anterior cingulate cortex activation during reward processing [31–34].

Comparisons of neural circuit function during reward processing in BD and MDD adults [29, 35, 36] and in high-risk studies [37, 38] motivate a need to understand the origins of these differences prior to any symptom onset. Indeed, symptomatic BD-risk youth have greater frontal-pole activation, decreased ventral striatum–VLPFC [37], lower ventral striatum–anterior cingulate, and greater pars orbitalis–orbitofrontal cortex functional connectivity compared with youth offspring of parents with MDD and other psychopathologies and healthy controls, even after removing the effects of symptoms and treatment [38]. To our knowledge, no studies have a priori compared neural reward function among healthy youth offspring with at least one parent with BD (BD-risk), healthy youth offspring with at least one parent with MDD (MDD-risk), and healthy offspring of families without first- or second-degree relatives with psychopathology (HC), during a never-symptomatic stage of relative psychological health. Understanding the differences among healthy at-risk groups may clarify the origins and pathophysiology of these overlapping but distinct conditions.

We previously described networks of regions implicated in differentiating BD risk from MDD risk during emotion processing [39], and neural markers of reward function in healthy offspring of parents with BD [25]. Here, we investigated reward processing in healthy offspring of parents with BD (BD-risk) and MDD (MDD risk), and healthy controls (HC) to determine whether distinct neural markers of reward function are present prior to symptom onset. We used the Monetary Incentive Delay Task [16, 25] to probe reward function. Based on previous studies [16, 25, 26, 29–38, 40], we examined differences in activation and connectivity in a priori regions of interest among BD-risk, MDD-risk, and HC youth. In response to reward anticipation, we hypothesized that BD-risk will exhibit decreased thalamic activation compared with MDD-risk and HC. Based on previous studies [37, 38], during outcome of rewards, we hypothesized that BD-risk will exhibit aberrant activation of prefrontal regions and reduced negative NAcc–VLPFC functional connectivity compared with MDD-risk and HC during reward anticipation and outcome. Since reward dysfunction in youth with mood disorders is associated with significant functional impairments over time [5, 41–43], we examined whether neural differences between BD-risk and MDD-risk were related to impulsivity, novelty-seeking, and behavioral strengths and difficulties, or conversion to psychopathology at longitudinal follow-up approximately 4.4 years after baseline.

## Methods

### Participants

Participants included 126 healthy youth between ages 8 and 17 years with no current or past Diagnostic and Statistical Manual of Mental Disorders (DSM–IV) Axis-I disorder. Forty had at least one parent diagnosed with bipolar-I disorder (BD risk), forty one had at least one parent diagnosed with major depressive disorder (MDD risk), and forty five had no personal or family history of psychopathology (HC). Youth were recruited from an academic mood-disorder program and the surrounding community. The Institutional Review Board approved the study, and written informed assent and consent were obtained from youth and parents, respectively, prior to study procedures. More details about participant inclusion and exclusion criteria are presented in Supplementary Methods.

### Assessment of psychiatric health

Participants were assessed using semistructured interviews by trained raters as described previously [39] and in Supplementary Methods. Youth were interviewed using the Children’s Depressive Rating Scale-Revised (CDRS-R) [44], Young Mania Rating Scale (YMRS) [45], and Multidimensional Anxiety Scale (MASC) [46] to confirm absent or low depression, mania, and anxiety-symptom severity, respectively.

Youth were followed longitudinally over 4.37 ± 2.29 (mean ± SD) years and evaluated for mood-symptom development. More details are described in the Supplementary Methods. At baseline, the Revised Dimensions of Temperament (DOTS-R) Survey [47], and Sensitivity to Punishment and Sensitivity to Reward Questionnaire (SPSRQ) for children [48] were completed by parents during euthymia. We focused on the DOTS-R approach-withdrawal score, which indexes the degree of novelty-seeking and on the SPSRQ impulsivity subscale, which measures levels of dysfunctional impulsivity. At baseline and follow-up, parents completed the Strengths and Difficulties Questionnaire (SDQ) [49], to assess psychosocial strengths (alluding to adaptive behaviors) using the Prosocial Behaviors SDQ subscale. Psychopathological difficulties (alluding to problem behaviors) were also assessed using a combined Total Difficulties SDQ subscale. All parents were euthymic at the time of assessment.

### Statistical analysis

We administered the Monetary Incentive Delay (MID) Task [50] during functional magnetic resonance imaging (fMRI) to participants. Task design, fMRI data acquisition, preprocessing, and statistical analyses including power estimation are detailed in the Supplementary Methods.

To examine group differences in neural activation during anticipation and outcome of monetary gain and loss, region-of-interest (ROI) analyses were conducted. A priori ROIs were selected based on regions activated in at-risk youth during reward processing, including the thalamus, VLPFC, NAcc, and putamen [51]. We conducted analyses of covariance (ANCOVA) for anticipation and outcome contrasts adjusting for gender, ethnicity, mean-centered age, CDRS-R, and YMRS scores (*p* < 0.05, false-discovery rate (FDR) corrected). Ethnicity was included as a covariate for all analyses due to group differences in ethnicity.

We conducted psychophysiological interaction (PPI) analyses with a whole-brain target mask and the thalamus as the seed region since this ROI exhibited a group difference during anticipation gain versus no gain. We examined group differences in context-dependent functional connectivity, covarying for gender, ethnicity, mean-centered age, CDRS-R, and YMRS scores (Z > 3.1, *p* < 0.05, family-wise error (FWE) cluster-corrected) [52].

We conducted voxelwise whole-brain analyses to evaluate other regions not included in our a priori hypotheses. Group comparisons were conducted with voxelwise whole-brain *F*-tests covarying for gender, ethnicity, mean-centered age, CDRS-R, and YMRS scores for anticipation and outcome contrasts (*Z* > 3.1, *p* < 0.05, FWE cluster-corrected) [52].

Preliminary and exploratory linear regressions within BD risk and MDD risk, covarying for age, gender, and ethnicity, were run to examine the associations between reward brain markers and novelty-seeking and impulsivity as assessed by the DOTS-R and SPSRQ and subsequent behavioral outcomes assessed by follow-up SDQ Total Difficulties and Prosocial scores.

Baseline SDQ scores were included as covariates for linear regressions using follow-up SDQ variables. We applied a false-discovery rate (FDR) correction for multiple tests to account for testing four regressions in each group. To account for variability in longitudinal follow-up and to explore the relations between neural findings and early stages of clinical conversion to a mood disorder, we ran cox regression analyses with age, gender, and ethnicity as covariates within BD risk and MDD risk. These exploratory analyses are included in the Supplementary Results.

Additional post hoc analyses, including analyses across both risk groups, age effects, and machine learning to predict group status, are included in the Supplementary Methods, Supplementary Results, and Supplementary Discussion.

## Results

### Participant demographics and clinical characteristics

From the original 151 participants included in this study, three BD-risk, six MDD-risk, and one HC youth were excluded due to not having complete or useable scans for the MID task. An additional four BD-risk, five MDD-risk, and six HC youth were excluded due to excessive head motion in the scanner. The final sample consisted of 40 BD-risk, 41 MDD-risk, and 45 HC youth.

Demographic, clinical, behavioral characteristics, parent diagnoses, and psychiatric diagnoses at follow-up are presented in Tables 1, 2, and Supplementary Table 1. There were no group differences in age, length of follow-up, IQ, CDRS scores, YMRS scores, motion artifact, or task accuracy and reaction time (all *p*s > 0.05). There was a group difference in ethnicity, *χ*^2^ (2, *N* = 126) = 20.77, *p* = 0.01. HC included more individuals who identified as Asian compared with BD risk and MDD risk. There were no differences among the groups on the DOTS-R approach-withdrawal score (*p* = 0.28) and SPSRQ impulsivity score (*p* = 0.52).

**Table 1:** Participant characteristics at baseline.

	BD-risk (*n* = 40)		MDD-risk (*n* = 41)		HC (*n* = 45)	*F* or *χ*^2^	*p*
Female (N)	26 (65%)		18 (43.9%)		27 (60.1%)	2.04	0.13^a^
Baseline age	12.47 (2.76)		13.56 (2.24)		13.21 (2.65)	1.91	0.15^b^
Age at follow-up	17.44 (4.08)		16.94 (2.63)		18.15 (2.98)	1.51	0.23^b^
Length of follow-up (years)	4.87 (2.47)		3.29 (1.11)		4.94 (2.60)	7.47	<0.01^b^
Intellectual quotient (IQ)	113.67 (10.79)		112.75 (14.42)		117.25 (13.26)	1.43	0.24^b^
Children’s depression rating scale-R (CDRS)	21.41 (6.59)		20.26 (3.93)		18.89 (2.52)	3.14	0.05^b^
Young mania rating scale (YMRS)	2.23 (3.34)		1.95 (3.82)		0.82 (1.28)	2.63	0.08^b^
Multidimensional anxiety scale (MASC)	38.17 (15.81)		40.541 (16.42)		35.370 (17.95)	0.74	0.48^b^
Children’s global assessment scale (CGAS)	87.11 (4.93)		87.32 (5.59)		91.25 (4.93)	8.56	<0.01^b^
Ethnicity, N (%)						20.77	0.01^a^
White or Caucasian	31 (78%)		27 (66%)		21(47%)		
Asian	1 (3%)		4 (10%)		15 (33%)		
Black or African American	0 (0%)		1 (2%)		1 (2%)		
Hispanic or Latino	1 (3%)		4 (10%)		3 (7%)		
Native American or Pacific Islander	0 (0%)		1 (2%)		0 (0%)		
Mixed race or other	7 (18%)		5 (12%)		5 (11%)		
*Parental diagnosis*							
	Mother	Father	Mother	Father			
*Bipolar disorder (BD)*	29	11	0	0	–	–	–
BD only	(14)	(6)	(0)	(0)			
BD + anxiety disorder	(4)	(1)	(0)	(0)			
BD + anxiety disorder + eating disorder	(1)	(0)	(0)	(0)			
BD + anxiety disorder + substance abuse	(3)	(0)	(0)	(0)			
BD + anxiety disorder + substance abuse + eating disorder	(1)	(0)	(0)	(0)			
BD + anxiety disorder + substance abuse + psychosis	(1)	(0)	(0)	(0)			
BD + anxiety disorder + ADHD	(1)	(0)	(0)	(0)			
BD + ADHD	(1)	(0)	(0)	(0)			
BD + eating disorder	(1)	(0)	(0)	(0)			
BD + psychosis	(1)	(0)	(0)	(0)			
BD + substance abuse	(1)	(4)	(0)	(0)			
*Major depressive disorder (MDD)*	0	4	28	17	–	–	–
MDD only	(0)	(2)	(20)	(12)			
MDD + anxiety disorder	(0)	(1)	(4)	(5)			
MDD + anxiety disorder + eating disorder	(0)	(0)	(3)	(0)			
MDD + anxiety disorder + substance abuse	(0)	(0)	(1)	(0)			
MDD + substance use	(0)	(1)	(0)	(0)			
*Dimensions of temperament survey-revised*							
Approach-withdrawal	20.03 (3.83)		19.11 (3.81)		18.64 (3.21)	1.31	0.28^b^
*The sensitivity to punishment and sensitivity to reward questionnaire (SPSRQ)*							
Impulsivity	1.99 (.62)		2.22 (.59)		2.16 (.75)	0.67	0.52^b^

*Note*. Values indicate the mean (SD) unless otherwise noted. ADHD attention-deficit/hyperactivity disorder, BD bipolar disorder, BD-risk youth at risk for bipolar disorder, F/U follow-up, GAD generalized anxiety disorder, HC healthy control youth, MDD major depressive disorder, MDD-risk youth at risk for a depressive disorder, OCD obsessive compulsive disorder, PTSD post-traumatic stress disorder.

^a^Statistic computed using *χ*^2^ test.

^b^Statistic computed using ANOVA.

**Table 2:** Participant diagnostic and behavioral characteristics at baseline and follow-up.

	BD-risk (*n* = 40)		MDD-risk (*n* = 41)		HC (*n* = 45)		*F*		*p*	
	Baseline	F/U	Baseline	F/U	Baseline	F/U	Baseline	F/U	Baseline	F/U
**Diagnosis**										
*Bipolar disorder (BD)*	0	1	0	0	0	0	–		–	
BD + substance abuse (cannabis) + panic disorder + ADHD	(0)	(1)	(0)	(0)	(0)	(0)				
*Major Depressive Disorder (MDD)*	0	7	0	13	0	4	–		–	
MDD only	(0)	(7)	(0)	(8)	(0)	(2)				
MDD + generalized anxiety disorder (GAD)	(0)	(0)	(0)	(4)	(0)	(2)				
MDD + substance abuse (cannabis)	(0)	(0)	(0)	(1)	(0)	(0)				
*Unspecified depressive disorder*	0	2	0	2	0	1	–		–	
*Generalized anxiety disorder*	0	3	0	2	0	2	–		–	
GAD only	(0)	(2)	(0)	(2)	(0)	(2)				
GAD + ADHD	(0)	(0)	(0)	(0)	(0)	(0)				
GAD + ADHD + OCD	(0)	(1)	(0)	(0)	(0)	(0)				
*Social Phobia*	0	1	0	0	0	(0)	–		–	
*Strengths and difficulties questionnaire*										
Prosocial scale	8.54 (1.56)	8.78 (1.70)	8.13 (2.14)	8.67 (1.61)	8.68 (1.34)	9.09 (1.44)	0.63	0.57	0.54	0.57
Total Difficulties	7.38 (4.48)	6.48 (4.18)	8.65 (5.44)	7.67 (4.51)	5.84 (4.31)	5.00 (2.65)	1.91	3.50	0.16	0.04

*Note*. Values indicate the Mean (SD) unless otherwise noted. ADHD attention-deficit/hyperactivity disorder, BD bipolar disorder, BD-risk youth at risk for bipolar disorder, F/U follow-up, GAD generalized anxiety disorder, HC healthy control youth, MDD major depressive disorder, MDD-risk youth at risk for a depressive disorder, OCD obsessive compulsive disorder, PTSD post-traumatic stress disorder. Statistic computed using ANOVA.

At baseline, there were no group differences in SDQ Prosocial and Total Difficulties subscales (all *p*s > 0.05). At follow-up, there were no group differences in the Prosocial subscale (*p* > 0.05). However, at follow-up, there was a group difference in Total Difficulties, *F*(2,82) = 3.50; *p* = 0.04, with the MDD-risk showing increased total difficulties compared with HC (post hoc *t*-test: *t*(54)= 2.77; *p* = 0.01). Further, the change from baseline to follow-up was significant for Total Difficulties (*F*(2,38) = 3.62; *p* = 0.04) but not for the Prosocial subscale (*p* > 0.05). Details about group differences during SDQ difficulties subscales: Emotional Problems, Peer Problems, Conduct Problems, and Hyperactivity are reported in Supplementary Table 2.

### ROI-analysis results for MID task

**Fig. 1:**
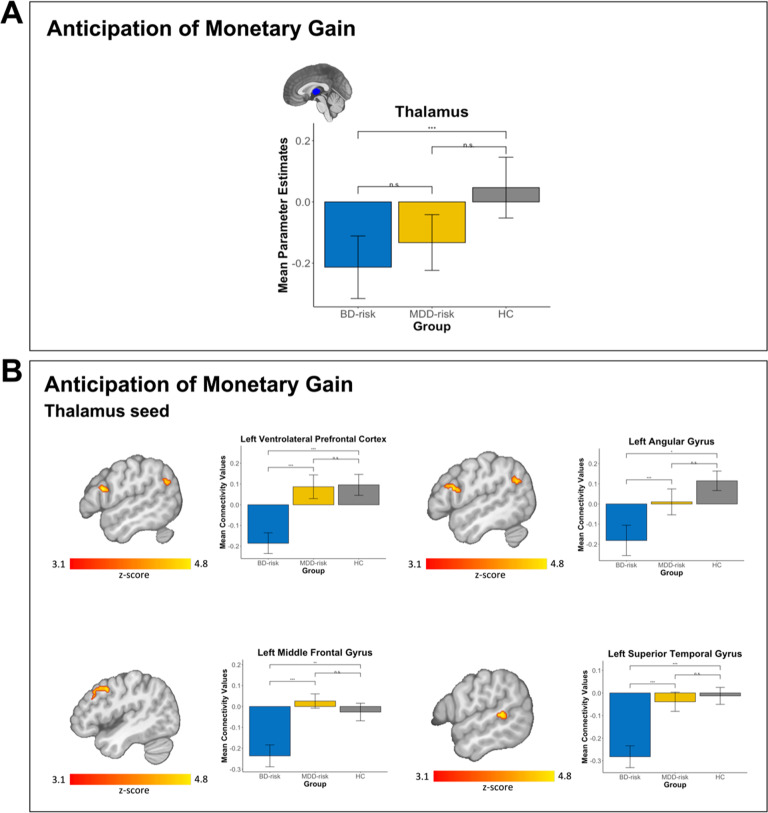
Significant group differences within regions of interest and functional connectivity during reward processing. **A** BD-risk had decreased activation in the thalamus compared with MDD-risk and HC during anticipation of a monetary gain versus anticipation of no monetary gain. **B** The BD-risk group had reduced connectivity between the thalamus and left VLPFC and left angular gyrus compared with the HC group, and reduced connectivity between the thalamus and left middle frontal gyrus and left superior temporal gyrus compared with the MDD-risk group during anticipation of monetary gain versus no monetary gain. Z-statistics images were thresholded (Z > 3.1) using corrected-cluster significance threshold of *p* < .05. Legend: blue: BD-risk, yellow: MDD-risk, gray: HC. Left side of the image corresponds to the left hemisphere. Error bars are standard errors of the mean. n.s. = not significant; *p < 0.05, **p < 0.01, ***p < 0.001.

During our ROI analysis, BD-risk had decreased activation in the thalamus compared with MDD-risk and HC during the anticipation of monetary gain versus no gain (*F*(2,118) = 4.64; *p* = 0.04, FDR-corrected), shown in Fig. 1A. There were no other significant differences in brain activation among the groups during anticipation of monetary loss versus no loss or in outcome conditions (FDR-corrected *p* > 0.05). ROI regions that did not survive FDR correction but were explored are depicted in Supplementary Fig. 1.

### Functional connectivity

During anticipation of monetary gain versus no gain, BD-risk had less connectivity between the thalamus and left VLPFC (*Z* > 3.1; *p* < 0.01) and left angular gyrus compared with HC (*Z* > 3.1; *p* < 0.05), shown in Fig. 1B and Table 3. BD-risk had less connectivity between the thalamus and left middle frontal gyrus (*Z* > 3.1; *p* < 0.001) and left superior temporal gyrus (*Z* > 3.1; *p* < 0.05) compared with MDD-risk, shown in Fig. 1B and Table 3. There were no other significant differences in functional connectivity among the groups during anticipation of monetary loss versus no loss or in outcome conditions (FDR-corrected *p* > 0.05).

**Table 3:** Significant seed-based and voxel-wise clusters of brain activation and connectivity.

								Peak MNI coordinates		
	Contrast	Peak brain region	Side	BA	Cluster size	Peak Z-score	*p*	x	y	z
HC > BD-risk	Thalamus seed Anticipation gain > Anticipation no gain	Ventrolateral Prefrontal cortex	L	44	121	4.58	<0.01	−46	22	20
HC > BD-risk	Thalamus seed Anticipation gain > Anticipation no gain	Angular gyrus	L	39	85	3.85	0.04	−52	−60	28
MDD-risk > BD-risk	Thalamus seed Anticipation gain > Anticipation no gain	Middle frontal gyrus/ dorsolateral prefrontal cortex	L	9	210	4.59	<0.001	−44	8	44
MDD-risk > BD-risk	Thalamus seed Anticipation gain > Anticipation no gain	Superior temporal gyrus	L	22	83	4.22	0.04	−58	−40	8
F test (BD-risk vs. MDD-risk vs. HC)	Anticipation gain > Anticipation no gain	Cerebellum	L	–	110	4.01	0.04	−4	−58	−8
F test (BD-risk vs. MDD-risk vs. HC)	Outcome monetary gain > Outcome no monetary gain or loss	Cerebellar crus II	R	–	131	4.37	0.01	22	−76	−44

*Note*. Coordinates are reported in Montreal Neurological Institute (MNI) space. BA Brodmann area, BD-risk youth at risk for bipolar disorder, HC healthy control youth, L left, MDD-risk youth at risk for major depressive disorder, R right.

### FMRI whole-brain voxelwise results for MID task

**Fig. 2:**
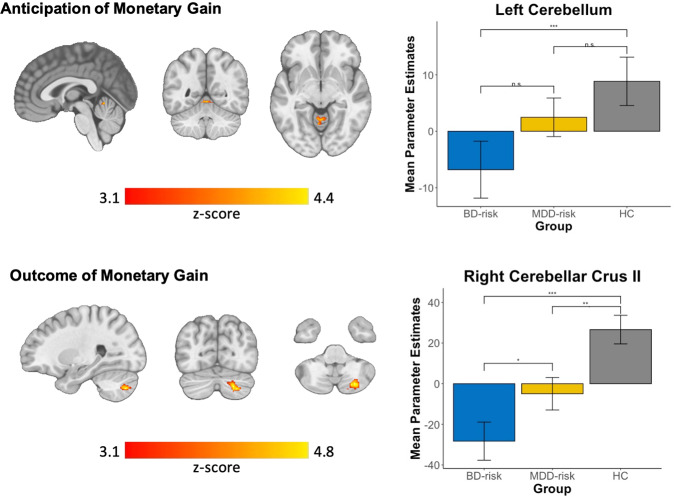
Significant group differences within whole-brain clusters during reward processing. BD-risk had reduced activation in the left cerebellum compared with MDD-risk and HC during anticipation of monetary gain versus anticipation of no monetary gain. BD-risk had reduced activation in the right cerebellar crus II compared with MDD-risk and HC during outcome of monetary gain versus outcome of no monetary gain or loss. Z-statistics images were thresholded (Z > 3.1) using corrected-cluster significance threshold of *p* < 0.05. Legend: blue: BD-risk; yellow: MDD-risk; gray: HC. Left side of the image corresponds to the left hemisphere. Error bars are standard errors of the mean. n.s. = not significant; *p < 0.05, **p < 0.01, ***p < 0.001.

BD-risk had decreased activation in the left cerebellum during anticipation of monetary gain versus no gain (*Z* > 3.1; *p* < 0.001) and decreased activation in the right cerebellar crus II during outcome of monetary gain versus no gain or loss (*Z* > 3.1; *p* < 0.01), shown in Fig. 2 and Table 3. There were no other significant whole-brain group differences in brain activation during anticipation of monetary loss versus no loss or outcome of monetary loss versus no gain or loss (*Z* > 3.1; *p* > 0.05).

### Relation between baseline reward processing and behavior at follow-up

Within BD-risk, decreased thalamus–angular gyrus connectivity during anticipation gain versus no gain was associated with increased impulsivity (*β* =−0.61; *p* = 0.04, FDR-corrected). Thalamus and left-superior temporal gyrus hypoconnectivity during anticipation gain versus no gain was associated with decreased follow-up SDQ Prosocial (*β* = 0.37; *p* = 0.04, FDR-corrected). Decreased cerebellum activation during anticipation gain versus no gain was associated with increased follow-up SDQ Total Difficulties (*β* = −0.56; *p* = 0.012, FDR-corrected). No other significant behavioral associations were observed between neural activation and connectivity and follow-up SDQ, approach-withdrawal, and impulsivity scores that survived multiple corrections in the BD-risk or MDD-risk groups (all *p*s > 0.05). Behavioral associations that did not survive FDR correction but were explored are reported in Supplementary Results.

## Discussion

This study identified neural markers of reward processing that distinguish healthy youth at familial risk for BD from healthy youth at familial risk for MDD and low-risk healthy-comparison youth at a stage of relative psychological health. BD-risk had decreased thalamus activation and hypoconnectivity between the thalamus and VLPFC, angular gyrus, middle frontal gyrus, and superior temporal gyrus, while anticipating monetary gain relative to MDD-risk and HC youth. Voxelwise, BD-risk youth had less activation in the cerebellum during anticipation of monetary gain and outcome of monetary gain relative to MDD-risk and HC. Within BD-risk, decreased thalamus–angular gyrus connectivity was associated with increased impulsivity at baseline, decreased thalamus–superior temporal gyrus connectivity was associated with decreased prosocial behavior at follow-up, and decreased cerebellar activation was associated with increased total difficulties at follow-up.

Reduced thalamic activation and connectivity during anticipation of gain may represent early and unique trait markers for BD-risk, or markers of risk for broader affective psychopathology [16]. The thalamus relays and integrates reward-related information flowing from subcortical to higher cortical areas, enabling encoding and modulation of salient, approach-related emotions during reward processing [53]. Reward-sensitivity dysfunction in BD-risk youth prior to symptom onset might originate from aberrant limbic and executive control-network function, which under typical circumstances, would recruit attentional control processes to support goal-directed behavior. The preliminary association between thalamic to angular gyrus hypoconnectivity during anticipation of gain and increased impulsivity merits replication. Unregulated perception and representation of goal value may heighten reward sensitivity and elevated attention impulsivity [54], increasing the risk for developing affective psychopathology, as supported by thalamus–left angular gyrus hypoconnectivity predicting increased risk of converting to a mood or anxiety disorder across both risk groups (Supplementary Results).

Thalamus–superior temporal gyrus hypoconnectivity in BD-risk youth was associated with decreased prosocial behaviors at, on average, 4-year follow-up. Lower prosocial behavior may be indicative of a higher likelihood of clinically significant social and behavioral problems in the future [55, 56]. Prosocial behaviors are commonly developed in adolescence, when both social development [57] and mania onset [3] are most acutely experienced. Thus, it is possible for decreased thalamic connectivity to be a vulnerability marker for future behavior problems in BD-risk youth. To our knowledge, these findings provide the earliest observation along the bipolar-risk continuum for thalamic network dysfunction and its relations to reward sensitivity, decision-making, and essential social behaviors at a time when mood symptoms frequently emerge [58]. Future studies could potentially consider targeting behaviors that improve thalamic function and connectivity during early psychosocial interventions in BD-risk youth who show signs of attentional impulsivity and problems with prosociality [59].

During anticipation and outcome of monetary gain, BD-risk youth demonstrated less activation in the left cerebellum and right cerebellar crus II, respectively, compared with MDD-risk and HC youth. The cerebellum, implicated in regulation, cognition, and affect [60], bidirectionally connects to prefrontal, parietal cortical areas, and limbic regions [61, 62], thereby regulating inhibition and reward learning [63, 64]. Aberrant cerebellar function in BD and BD risk [65–69] may contribute to bipolar-symptom expression [70–73]. Behaviorally, decreased cerebellar activation correlated with increased propensity toward psychiatric dysfunction as measured by follow-up SDQ total difficulties among BD-risk youth. Understanding the role of the cerebellum in reward function in bipolar disorder may clarify the significance of our voxelwise observations.

Family studies provide evidence for distinct familial aggregation patterns in mania and depression [11]. Because BD-risk youth may be at risk for other, non-BD disorders and MDD-risk youth may be at risk for BD, we evaluated risk markers of reward processing for affective psychopathology by combining the high-risk groups and comparing them to HC. ROI and whole-brain analyses revealed that high-risk youth had decreased activation in the putamen, ventrolateral prefrontal cortex, and cerebellar crus compared with HC during outcome of monetary gain (Supplementary Fig. 2). These regions have been previously associated with functional abnormalities in MDD and BD [25, 37, 63, 64]. Thalamic activation did not survive multiple-comparison corrections when BD risk and MDD risk were combined (Supplementary Fig. 3), suggesting that it may be uniquely critical for reward function in BD risk.

Our study is the largest to compare neural markers of risk in healthy youth at familial risk for BD and MDD in relation to subsequent behavioral and psychiatric outcomes. Nevertheless, we acknowledge several limitations of this work. Neuroimaging data were collected at one time point, which precludes determination of whether reward circuitry differences between BD risk and MDD risk represent neural vulnerability markers or compensatory adaptation to familial risk. Future studies should evaluate neural markers longitudinally to examine changes in brain activation and connectivity over time and delineate neural biomarkers specific to BD risk versus MDD risk. A relatively wide age range of youth participated and though there were no significant age differences between groups or interactions between age and group, differential patterns of activation within other reward regions may undergo nuanced changes during sensitive windows that are obscured through group averages. With an expanded dataset, the wide age range may provide future opportunity to granularly explore critical sensitive subwindows for mood-disorder conversion. Finally, measures related to depression, mania, and anxiety were assessed at baseline to establish asymptomatic status for inclusion and are continuing to be collected over longitudinal follow-up with nonclinical or subclinical values in a subset of the sample. This precludes us from examining associations between neural markers and change in psychiatric symptoms from an asymptomatic baseline to follow-up across all participants. Future studies that evaluate dimensional mood-symptom changes longitudinally may be able to delineate neural biomarkers of BD risk relative to MDD risk while mapping symptom development. Further, future studies could employ machine-learning techniques to determine if neural correlates predict risk-group membership (see our preliminary results: Supplementary Table 3, Supplementary Fig. 4).

Our study highlights potential differential vulnerabilities for BD risk compared with MDD risk that are well contextualized in studies that differentiate these disorders when the syndromes are fully expressed. Elucidating unique neural, behavioral, and clinical predictors of future reward dysregulation is a step forward toward identifying objective markers of BD risk and may provide selective targets to better guide prevention and early interventions in youth with and at risk for mood disorders. Supplementary information is available at TP’s website.

## Supplementary information

Supplementary Information
