# Supplementary material for: Neural correlates of reward processing distinguish healthy youth at familial risk for bipolar disorder from youth at familial risk for major depressive disorder
Source: Transl Psychiatry. 2022 Jan 24;12:31. doi: 10.1038/s41398-022-01800-9 (PMC8786954; doi:10.1038/s41398-022-01800-9)
Supplement: Supplementary file 1 — Supplementary Information [file 41398_2022_1800_MOESM1_ESM.doc]

**Supplementary Information**

**Neural Correlates of Reward Processing Distinguish Healthy Youth at Familial Risk for Bipolar Disorder from Youth at Familial Risk for Major Depressive Disorder**

Akua F. Nimarko, PhD, Aaron J. Gorelik, BS, Kayla E. Carta, BS, Mark G. Gorelik, BS, Manpreet K. Singh, MD, MS

**Contents**

**Supplementary Methods**

**Supplementary Results**

**Supplementary Discussion**

**Supplementary Table 1.** Task and Motion Performance

**Supplementary Table 2.** Participant SDQ Difficulties Characteristics

**Supplementary Table 3.** Feature Sets

**Supplementary Figure 1.** Group Differences Within Region of Interests During Reward Processing

**Supplementary Figure 2.** Significant High-Risk Group Differences Within Region of Interests and Whole-brain Clusters During Reward Processing

**Supplementary Figure 3.** High-Risk Group Differences Within Region of Interests During Reward Processing

**Supplementary Figure 4.** Feature Set Accuracy and Permutation Feature Importance

**References**

**Supplementary Methods**

*Participants*

Exclusion criteria for all groups at baseline included: 1) currently taking medication or receiving psychotherapy for any psychiatric disorder; 2) having a current or lifetime diagnosis of any psychiatric or substance use disorders, or neurological disorders; 3) MRI contraindications including orthodontic braces; or 4) intellectual quotient (IQ) <80 assessed by the Weschler Abbreviated Scale of Intelligence (WASI) [1]. Ethnicity was defined by participants and collected for the purpose of reporting participant demographic data.

*Assessment of Psychiatric Health*

Structured diagnostic interviews were administered separately to participants and their parents (regarding the participants) using the mood sections of the Washington University in St. Louis Kiddie-Schedule for Affective Disorders and Schizophrenia (WASH-U KSADS) [2], and the Kiddie Schedule for Affective Disorders and Schizophrenia for School-Age Children-Present and Lifetime version (KSADS-PL) for all other psychopathology [3]. The Structural Clinical Interview for the DSM-IV (SCID-IV) [4] was administered to parents to confirm parental diagnosis of a major mood and other psychiatric disorders. All diagnoses were reviewed and confirmed by a board-certified child and adolescent psychiatrist (MKS).

*Follow-up Visit*

Participants either came in for an in-person follow-up visit or had a phone follow-up interview. During the in-person follow-up visit, structured diagnostic interviews were administered separately to participants and their parents (regarding the participants, if were under the age 18) using the mood sections of the Washington University in St. Louis Kiddie-Schedule for Affective Disorders and Schizophrenia (WASH-U KSADS) [2], and the Kiddie Schedule for Affective Disorders and Schizophrenia for School-Age Children-Present and Lifetime version (KSADS-PL) for all other psychopathology [3]. All diagnoses were reviewed and confirmed by a board-certified child and adolescent psychiatrist (MKS).

During the phone follow-up interviews, participants were asked to report if they had received a diagnosis for a psychiatric disorder since the baseline visit and were also given assessments to assess for mood symptom development. Youth who met diagnostic criteria for a psychiatric disorder were classified as converted, and youth who did not report any symptoms were classified as resilient. All diagnoses were reviewed and confirmed by a board-certified child and adolescent psychiatrist (MKS).

*Strengths and Difficulties Questionnaire*

Psychopathological difficulties of participants through the subscales Emotional Problems, Peer Problems, Conduct Problems, and Hyperactivity on the Strenghts and Difficulties Questionnaire (SDQ) [5] were assessed by parents during euthymia at both baseline and follow-up.

*Monetary Incentive Delay (MID) Task*

Prior to the scan, all participants underwent a practice MID task to test for comprehension of explicit cues and assess individual differences in reaction times to cue responses. Participants were also presented with potential prizes that they could win if they earned enough money as previously described. Participants completed two runs of the MID task which were counterbalanced to control for practice effects. The MID task was designed to evaluate neural responses to anticipation and receipt of gain and avoiding loss conditions, using a set of cues to indicate whether participants can win or avoid losing money if they respond quickly enough to a target (represented by a triangle) that follows a cue and anticipation period. Circle cues indicate a chance to gain money whereas square cues represented opportunities for loss. These cues are marked with either 0, 1, or 2 horizontal lines to represent the values of the gain or loss condition: $0, $1, and $5 respectively. Altogether, there are six possible cues. During each trial, a cue is presented for 250 msec followed by a jittered anticipatory delay period (2000-2500 msec) and then presentation of a target (250-350 msec). If the participant pressed a button in response to the target quickly enough, then they gain money or avoid loss. Failure to respond in time results in no gain of money or loss. The target duration for each patient was based on their reaction time in order to achieve an approximately 66% success rate. Following offset of the target screen, a jittered delay period came before outcome to ensure total trial length was 6 seconds. The outcome screen was displayed for 1650 msec to show participants whether they responded in time. There is a total of 54 trials per run with each of the six cues appearing 9 times pseudo-randomly. At the end of task, we tested participant comprehension of the test and asked about them to subjectively rate their emotional valence and arousal towards each cue.

*Neuroimaging Data Acquisition*

Before the MRI scan, participants were familiarized with the scan environment and trained to minimize head motion using a mock scanning procedure. MRI images were acquired on a 3 Tesla GE Signa scanner (General Electric Co., Milwaukee, WI) using an 8-channel head coil. High resolution anatomical images were acquired to optimize normalization of functional images to a standard template (3D FSPGR pulse sequence; TR = 8.5 ms, TE = 3.32 ms, TI = 400 ms, flip angle = 15°, field of view = 25.6 cm, 186 slices in the axial plane, resolution=1 cubic mm. Functional images were collected using a T2-weighted spiral in-out pulse sequence [6] with the following parameters: repetition time (TR) = 2000 ms, echo time (TE) = 30 , flip angle (FA) = 80°, field of view (FOV) =  22 cm, voxel-size 3.43 mm x 3.43 mm x 4 mm with 1 mm skip, number of slices = 30 slices in axial plane. A high-order shim was used to improve field homogeneity.

*Neuroimaging Preprocessing Steps*

Preprocessing in the fMRI Expert Analysis Tool (FEAT) function in FMRIB's Software Library (FSL) [7] included slice-timing correction, motion correction with MCFLIRT, brain extraction, smoothing with a Gaussian filter (5 mm full width at half-maximum) and volume registration to MNI152 standard-space [8]. Participants were excluded due to motion if their absolute or relative mean displacements exceeded 2 mm or more than one-fourth of volumes had values for spatial standard deviation of successive difference images (DVARS) exceeding the threshold of the 75^th^ percentile plus 1.5 times the interquartile range. Individual participants’ first level analyses were performed using FEAT in FSL. The two independent runs of the MID task were combined at a second level of analysis using a fixed effects model that allowed for within-subject, multi-session analyses.

*Region of Interest Analysis*

To examine group differences in neural activation during the anticipation and outcome of monetary gain and loss, a region of interest (ROI) analysis was conducted. *A priori* ROIs were selected based on regions known to be activated in at-risk youth during reward processing, including thalamus, ventrolateral prefrontal cortex (VLPFC), nucleus accumbens (NAcc), and putamen [9]. ROIs were anatomically defined using FSL’s Harvard-Oxford Subcortical Structural Atlas. Parameter estimates from these selected ROIs were extracted from each participant’s whole-brain map for each contrast. Analyses of covariance (ANCOVA) covarying for sex, ethnicity, mean-centered age, mean-centered CDRS-R, and mean-centered YMRS scores were conducted to examine group differences (BD-risk vs MDD-risk vs HC), for the following contrasts: anticipation gain total > anticipation no gain total, anticipation loss total > anticipation no loss total, outcome gain total hit > outcome no gain loss total hit, outcome gain total miss > outcome no gain loss total miss, outcome loss total hit > outcome no gain loss total hit, outcome loss total miss > outcome no gain loss total miss. Analyses of covariance (ANCOVA) were also conducted for the above contrasts with sex, ethnicity, mean-centered age, mean-centered CDRS-R, and mean-centered YMRS scores as covariates to examine group differences between High-Risk (combining BD-risk and MDD-risk) vs HC. We applied a false discovery rate (FDR) correction for multiple tests to account for testing four ROIs in each group. *Post hoc* t-tests were then conducted to determine which groups were driving the significant group differences.

*Whole-brain fMRI Data Analysis*

We conducted voxel-wise whole brain analyses to evaluate other regions not included in our a priori hypotheses. Functional data were processed using FSL. For individual subject statistical maps, the timeseries of each voxel was modeled with a general linear model (GLM). Trials presenting anticipation of monetary gain (i.e., $1 and $5) were combined to increase statistical power, as were trials presenting anticipation of monetary loss, outcome of successful monetary gain, outcome of unsuccessful monetary gain, outcome of successful monetary loss, and outcome of unsuccessful monetary loss. Anticipation of monetary gain, anticipation of monetary loss, outcome of successful monetary gain, outcome of unsuccessful monetary gain, outcome of successful monetary loss, and outcome of unsuccessful monetary loss conditions were modeled as regressors of interest. Target fixation and 24 motion correction parameters were included as regressors of non-interest. We also included time points that exceeded the DVARS motion threshold of 75th percentile plus 1.5 times the interquartile range as regressors of non-interest to remove the effects of these timepoints in our analyses [7]. Group comparisons were conducted with voxel-wise whole-brain F-tests using FSL’s FLAME (FMRIB’s analysis of mixed effects)[10] covarying for sex, ethnicity, mean-centered age, mean-centered CDRS-R, and mean-centered YMRS scores for the same contrasts mentioned above. Voxel-wise whole-brain t-tests were also conducted using FSL’s FLAME (FMRIB’s analysis of mixed effects) [10] with sex, ethnicity, mean-centered age, mean-centered CDRS-R, and mean-centered YMRS scores as covariates for the above contrasts to examine group differences between High-Risk and HC. Statistically significant clusters were identified by thresholding *Z*-statistic images (*Z*  > 3.1) with a FWE-cluster corrected probability of *p* < .05 [11]. We extracted mean parameter estimates from significant clusters using fslmeants to determine the direction of activation differences.

*Psychophysiological interaction (PPI) Analyses*

Psychophysiological interaction (PPI) analyses were conducted in FEAT to examine group differences in context-dependent functional connectivity associated with anticipating loss versus non-loss based on results from ROI results. Specifically, PPI analyses were conducted using the thalamus as a seed region of interest (ROI) because it exhibited a significant group difference during anticipation gain versus anticipation no gain within our primary fMRI ROI analysis. The thalamus seed was transformed into individual subject space to create masks and mean temporally filtered timeseries for each individual were then extracted. For our GLM analysis, the time course for the seed region was entered as a regressor of interest in addition to the main effect of task regressor and an interaction regressor. The interaction regressor was computed as the product of the mean extracted timeseries for the seed ROI and our contrast of interest, anticipation gain versus no gain. We also included the other task regressors, anticipation loss, anticipation no loss, outcome gain hit, outcome gain miss, outcome loss hit outcome loss miss, and target fixation and 24 motion correction parameters as regressors of non-interest. We included time points that exceeded the DVARS motion threshold calculated as regressors of non-interest to remove the effects of these timepoints in our analyses. Using a whole brain target mask, group comparisons were conducted using FLAME in FSL, covarying for sex, mean-centered age, and mean-centered CDRS-R scores, and mean-centered YMRS scores. Significant group differences were identified using a threshold of *Z* > 3.1 with a FWE-cluster corrected significant threshold of *p* < .05 [11]. Mean parameter estimates from significant PPI clusters were then extracted using fslmeants to determine the direction of group differences.

*Statistical Analyses of Clinical and Behavioral Data*

Follow-data was not collected for four participants in the BD-risk group and one participant in the MDD-risk group. All statistical analyses were conducted using SPSS Version 27 and R Version 3.6 and a power calculation was conducted in G*Power 3.1 to calculate effect sizes for our sample of 126 participants. We conducted one-way analyses of variance (ANOVAs) to test for a main effect of group in clinical and behavioral characteristics based on scores from the WASI, CDRS-R, YMRS, and CGAS. We also conducted repeated measures ANOVAs to test for a main effect of group on change from baseline to follow-up on the SDQ.

We also ran Pearson Correlation to test for any significant correlations between baseline brain activation and connectivity and CDRS, YMRS, and MASC within the BD-risk and MDD-risk groups, and within the High-Risk and HC groups. We tested for any significant between group correlations based on Fisher z-transformation.

We performed secondary hypothesis-generating linear regression analyses within the BD-risk and MDD-risk groups incorporating age, gender, and ethnicity, as covariates to test for associations between brain activation and connectivity and novelty-seeking and impulsivity as assessed by the DOTS-R and SPSRQ at baseline, to understand whether there were any associations between reward function and constructs of novelty-seeking and impulsivity. We also conducted exploratory linear regression analyses within the BD-risk and MDD-risk groups incorporating age, gender, ethnicity, and baseline SDQ scores as covariates to explore whether activation or connectivity within regions were associated with behavioral outcomes as assessed by SDQ scores at follow-up. We applied a false discovery rate (FDR) correction for multiple tests to account for testing four regressions in each group. Further, to account for variability in longitudinal follow-up, we ran cox regression analyses with age, gender, and ethnicity as covariates within the BD-risk and MDD-risk groups to examine whether brain region activation and connectivity starting from a stage of health was associated with a greater risk of conversion to a mood disorder (variables did not violate linearity in the logit or proportional hazards).

We also performed the same linear regression analyses mentioned above within the High-Risk group (combining BD-risk and MDD-risk). To account for variability in longitudinal follow-up and to explore relations between neural findings and early stages of clinical conversion to a mood disorder, we ran cox regression analyses with age, gender, and ethnicity as covariates within BD-risk and MDD-risk.

*Support Vector Machine (SVM) and Random Forest*

We explored the use of machine learning (ML) techniques to evaluate whether neural correlates during reward processing with additional features could predict risk group membership. Based on previous studies that have shown success with classifying fMRI-based data with limited sample datasets, two machine learning techniques were evaluated: Support Vector Machine (SVM) and Random Forest [12–19]. Using SciKit Learn based implementations, two feature sets of interest were computationally defined based on the significant brain differences in our primary analyses, behavioral measures, impulsivity and approach-withdrawal, and the covariates from our primary analyses: Feature Set 1: Ethnicity, Gender, Age, CDRS, YMRS, Impulsivity, Approach-withdrawal, Thalamus Activation, Thalamus- Left VLPFC Functional Connectivity (FC), Thalamus- Left Angular Gyrus FC, Thalamus- Left Middle Frontal Gyrus FC, and Thalamus- Left Superior Temporal Gyrus FC. Feature Set 2: Ethnicity, Gender, Age, CDRS, YMRS, Impulsivity, Approach-Withdrawal, Left Cerebellum Activation, and Right Cerebellar Crus II Activation. The defining difference between each feature set was the neural data used (see **Supplementary Table 4** for feature set breakdown). For each feature set of interest, a grid search was used to optimize the hyperparameters on a subset (80%) of the randomly selected data for each ML technique. The accuracy of each ML technique and feature set combination was measured with the optimized hyperparameters by creating over 200 random shuffled splits of the data (80%/20% train/test split). Using these measurements, the average accuracy and standard deviation of the ML technique was determined with selected hyperparameters and features with the confidence interval at p99. Permutation feature importance [14] was also used to evaluate the importance of each feature using accuracy as the metric. In all cases the same random seed value of 42 was used.

**Supplementary Results**

*Power Analysis*

A power analysis was performed using G*Power 3.1 to determine anticipated effect sizes for our analyses given our sample size of n=126. For our ANCOVA analyses, the required effect size is .35. For our linear regression analyses within the BD-risk group, the required effect size is .35 and within the MDD-risk group, the required effect size is .34. Based on our study design, ANCOVA analyses with an alpha = .05 and desired effect size = .80 would need a projected sample size of n=29. For linear regression analyses, with alpha = .05 and desired effect size = .80, a projected sample size of 36 would be needed. Thus, our total sample size (n=126), sample size for BD-risk (n=40), and MDD-risk (n=41) were adequate for examining the main objectives of this study.

*Tests for Normality*

We performed Kolmogorov-Smirnov Tests for normality for our main behavioral outcome measures. Approach-withdrawal, Impulsivity, and Follow-up Total Difficulties were normally distributed (*p*s > .05). Follow-up Prosocial scores did not follow a normal distribution, *D*(39)=.20, *p* = .03, and were slightly skewed to the right. Studies have suggested if the residuals in a linear regression are well behaved, “normality assumption” does not have to be completely met [20]. Nevertheless, these linear regressions are preliminary and we interpret the results cautiously with these considerations in mind.

*Tests of Homogeneity of Variances*

We performed Levene’s Tests for Equality of Variances to examine any significant differences in variance of activation and parameter estimates from significant regions from our main group analyses (thalamus, thalamus-left ventrolateral prefrontal cortex, thalamus-left angular gyrus, thalamus-left middle frontal gyrus, thalamus-left superior temporal gyrus, left cerebellum, and right cerebellar crus II). There were no significant differences in variance between the groups (all *p*s > .05).

*Age Interaction and Moderation Analyses*

We performed linear regression analyses with group, age, and group*age as independent variables and activation parameter estimates from significant regions from our main group analyses (thalamus, thalamus-left ventrolateral prefrontal cortex, thalamus-left angular gyrus, thalamus-left middle frontal gyrus, thalamus-left superior temporal gyrus, left cerebellum, and right cerebellar crus II) and High-risk group analyses (putamen, ventrolateral prefrontal cortex, and right cerebellar crus II) as dependent variables. There was an interaction effect between age and group for thalamus-left angular gyrus connectivity during anticipation gain > anticipation no gain (β = .04; *p* = .03); however, this did not survive multiple comparisons. There were no additional significant interaction effects between age and group (all *p*s > .05).

We also performed linear regression analyses with our behavioral measures as dependent variables and baseline neural activity and connectivity with group, age, and group*age as independent variables to test for moderating effects of age on neural-behavioral measure relationships both within the BD-risk and MDD-risk groups, and across the at-risk groups. There were no significant moderating effects of age (all *p*s > .05).

*Monetary Incentive Delay (MID) Task Performance*

There were no significant differences in task accuracy or reaction time among the BD-risk, MDD-risk, and HC groups (all *p*s > 0.05; **Supplementary Table 1**).

*Strengths and Difficulties Questionnaire (SDQ)*

Participant Difficulties SDQ characteristics are presented in **Supplementary Table 2**. There was a significant group difference for baseline SDQ Hyperactivity, *F*(2,63) = 3.39; *p* =.04 and follow-up SDQ Peer Problems, *F*(2,82) = 4.08; *p* =.02. There were no other group differences at baseline or follow-up. There were also no significant group differences in change from baseline to follow-up on other SDQ subscales (all *p*s > .05).

*Additional Exploratory ROI results for MID Task that did not survive FDR correction*

The BD-risk group had increased activation in the putamen compared to MDD-risk and HC groups during the anticipation of avoiding monetary loss versus no loss *F*(2,118) = 3.77; *p* =.03 shown in **Supplementary Figure 1**. The BD-risk group had increased activation in the thalamus compared to MDD-risk and HC groups during the anticipation of avoiding monetary loss versus no loss *F*(2,118) = 3.96; *p* =.02), shown in **Supplementary Figure 1**. Lastly, the BD-risk group had decreased activation in the VLPFC compared to the MDD-risk and HC groups during the outcome of monetary gain versus no monetary gain or loss *F*(2,110) = 3.87; *p* =.02), shown in **Supplementary Figure 1.** There were no other differences in brain activation among the groups during the other outcome conditions (*p* > .05). Further, there were no group differences in connectivity of the putamen, thalamus, and VLPFC during anticipation of monetary loss versus anticipation of no monetary loss and outcome of monetary loss versus outcome of no monetary reward or loss (*Z* > 3.1; *p* > .05).

*High-Risk Analyses Results for MID Task*

In the ROI analysis, the High-Risk group had decreased activation in the putamen *F*(1,111) = 6.32; *p* =.01 (FDR-corrected *p* = .03) and ventrolateral prefrontal cortex *F*(1,111) = 7.65; *p* =.007 (FDR-corrected *p* = .03) compared to HC during the outcome of monetary gain verses no gain, shown in **Supplementary Figure 2**. Our whole-brain analyses demonstrated reduced right cerebellar crus [k=297 voxels, peak x/y/z MNI coordinate=22/-76/-44, z=4.86, *p*= 0.000114], and left ventrolateral prefrontal cortex [k=139 voxels, peak x/y/z MNI coordinate=-48/24/0, z=4.32, *p*= 0.0114, BA45] activation during outcome of monetary gain in the High-Risk group compared to HC, shown in **Supplementary Figure 2**. There were no other significant differences in brain activation among the groups during anticipation of monetary gain versus no gain, anticipation of monetary loss versus no loss, and the other feedback conditions (FDR-corrected *p* > .05).

We also explored ROI regions that did not survive FDR-correction. High-Risk had decreased activation in the thalamus *F*(1,119) = 6.297; *p* =.014), and ventrolateral prefrontal cortex *F*(1,119) = 4.40; *p* =.04), compared to HC during the anticipation of monetary gain versus anticipation of no monetary shown in **Supplementary Figure 3.**

*Exploratory Relations among Baseline Neural Reward Processing and Nonclinical Symptoms*

There were no significant correlations between baseline neural markers, CDRS, YMRS and MASC within the BD-risk and MDD-risk groups and across both high-risk groups.

*Additional Exploratory Relations among Baseline Neural Reward Processing and Behavior that did not Survive FDR Correction.*

Within the BD-risk group, decreased right cerebellar crus II activation during outcome of monetary gain versus no monetary gain was associated with increased approach-withdrawal (*β* = -.04; *p* = .029). Decreased cerebellum activation during anticipation gain versus no gain was associated with increased SDQ emotional problems at follow-up (*β* = -.30; *p* =.02). Decreased thalamus activation during anticipation gain versus no gain was associated with increased SDQ peer problems at follow-up (*β* = -1.44; *p* <.05).

Within the MDD-risk group, decreased left cerebellum during anticipation gain versus no gain was associated with increased approach-withdrawal (*β* = -.13; *p* = .017) and increased left cerebellum during anticipation gain versus no gain was associated with increased follow-up SDQ Prosocial scores (*β* = .10; *p* = .04). There were no significant associations between brain activation and connectivity and follow-up SCQ scores within the MDD-risk group (all *ps* <.05).

*Exploratory Relations among Baseline Neural Reward Processing Behavior, and Conversion at Follow-Up Across High-Risk*

Across both risk groups, thalamus activation (*β* = 2.57; *p* = .035), and VLPFC activation (*β* = 2.06; *p* = .047) were positively associated with approach-withdrawal, and Thalamus-VLPFC connectivity (*β* = -2.69; *p* = .028) was negatively associated with approach-withdrawal during anticipation monetary gain > anticipation no gain. Right cerebellar crus II activation during outcome of monetary gain > outcome of no monetary gain or loss was negatively associated with approach-withdrawal (*β* = -.021; *p* = .002). Left VLPFC activation during outcome of monetary gain > outcome of no monetary gain or loss was positively associated with follow-up hyperactivity (*β* = .017; *p* = .015). Putamen activation was negatively associated with total difficulties at follow-up (*β* = -4.04; *p* = .01) and VLPFC activation was positively associated with total difficulties at follow-up (*β* = 1.24; *p* = .03) during outcome of monetary gain > outcome of no gain no loss.

Our cox regression models demonstrated that across the risk groups one standard deviation unit decrease in thalamus-left angular gyrus connectivity (HR = 1.85, 95% CI: [1.08-3.23], *p* = .03) was associated with an increased risk of converting to a mood or anxiety disorder.

*Relation Between Baseline Reward Processing and Conversion at Follow-Up*

Our cox regression models demonstrated that within MDD-risk, a one standard deviation unit decrease in thalamus-left middle frontal gyrus connectivity (HR=4.98, 95% CI: [1.042-23.79], *p* = .04) and one standard deviation unit decrease in right cerebellar crus II activation (HR= 7.14, 95% CI: [1.54-34.48], *p* = .01) were associated with an increased risk of converting to a mood or anxiety disorder. There were no associations between connectivity or activation and risk of conversion within the BD-risk group.

*Feature Performance*

Feature contributions for each ML technique and feature set combination are visualized with heatmaps in **Supplementary Figure 4.** The best performing ML technique was a random forest with an average accuracy of 63.7% (SD 8%) using Feature Set 2. This set of features was also the most effective for the SVM with an average accuracy of 55.2% (SD 8.7%). The random forest trained on feature set 1 performed with an average accuracy of 52.1% (SD 10%) and the SVM performed with an average accuracy 54% (SD 8.9%).

  For random forest Feature Set 1, Impulsivity, Thalamus- Left Superior Temporal Gyrus FC, and Thalamus- Left VLPFC FC contributed the most to the predictive power of both model types. In the case of random forest, removing them caused a mean accuracy decrease of 10.4%, 1.8%, 1.7% respectively. In the SVM the top three contributing features were Impulsivity, Ethnicity, and Thalamus Activation which caused a mean accuracy decrease of 12.8%, 5.8%, 3.5%. For Feature Set 2 in the case of random forest, removing Right Cerebellar Crus II Activation, Impulsivity and Left Cerebellum Activation caused a mean accuracy decrease of 25.4%, 18.4%, and 15.6% respectively. In the SVM removing Impulsivity, Right Cerebellar Crus II Activation, and Gender caused a mean accuracy decrease of 17.7% ,14.4%, and 9.4% respectively.

**Supplementary Discussion**

During anticipation of avoiding monetary loss, the BD-risk youth demonstrated increased putamen activation compared to MDD-risk and HC youth, suggestive of a unique biological marker for familial risk to BD. The putamen, located within the dorsal striatum, is critically involved in reward processing and relaying information about the salience of rewarding stimuli to direct motivation and goal-directed behavior, in particular to reinforce learning [21, 22]. Increased putamen activation when avoiding monetary loss in the BD-risk youth suggests a selective awareness of rewards that could be indicative of heightened hedonic symptoms such as increased pleasure-seeking activity typically seen in individuals with BD [23]. Thus, increased putamen activation in high-risk youth may represent a neurobiological vulnerability which predisposes high-risk children to impaired hedonic function. Further, we found blunted putamen activation in the MDD-risk youth, which has been previously reported in youth at familial risk for MDD [24, 25]. Therefore, the deactivation of the putamen observed within the MDD-risk youth could indicate decreased sensitivity to reward which is has been consistently seen in individuals with MDD [26, 27].

During anticipation of avoiding monetary loss, the BD-risk youth had significantly increased thalamus activation compared to MDD-risk and HC youth. The thalamus relays and integrates information flow from reward and higher cortical areas, enabling modulation and encoding of salient, approach-related emotions during reward processing [28]. Thus, abnormal thalamic activation during the anticipation of monetary loss could indicate that BD-risk youth have disordered insight during the avoidance of punishment.

During outcome of monetary gain, the BD-risk youth had decreased VLPFC activation compared to MDD-risk and HC youth. Abnormal VLPFC function in youth with and at-risk for BD have been documented [29–32], suggesting that VLPFC may be a trait-specific marker for BD. The VLPFC has been interpreted as serving an emotion regulatory function during reward processing by possibly modulating approach-related emotions during reward processing [33, 34]. Further, the VLPFC may encode the value of choice/decision-making options and assigned value to rewards [35]. Prior studies have also shown that impairments to the​ VLPFC ​mediated neural circuitry could underlie the cognitive and emotional deficits associated with abnormal reward processing in BD and other mood disorders [36, 37]. Thus, there is a possibility that abnormal function of the VLPFC during the outcome of reward in the BD-risk youth could represent dysfunction in the interpretation of positive reward and thus could be related to goal attainment that may manifest in BD as grandiosity and dysregulated goal pursuit.

We found associations between cerebellar and thalamic activation with novelty-seeking and emotional and peer problems at follow-up within the BD-risk youth, and cerebellar activation and novelty-seeking and prosocial behavior within the MDD-risk youth. Although speculative, these findings suggest that these brain abnormalities observed in the high-risk youth could subserve behavioral difficulties typically seen in youth with mood disorder [38–42].

Our cox regressions did not show evidence of baseline imaging predicting mood or anxiety disorder conversion within BD-risk. Rather, decreased thalamus-left middle frontal gyrus connectivity and right cerebellar crus II activation was associated with increased risk of converting to a mood or anxiety disorder within MDD-risk. This finding points to differential predictive sensitivity among MDD-risk over BD-risk youth [43, 44], or may be a function of an underpowered outcome.

Lastly, machine learning (ML) models, SVM and Random Forest, were able to accurately predict risk group membership based on cerebellum activation, thalamus activation and connectivity, impulsivity, and approach-withdrawal beyond chance. These findings suggest that BD-risk and MDD-risk youth may present with a combination of specific neural signatures and behaviors related to reward processing that are common and distinct. Differential activation and connectivity of the thalamus and cerebellum could be specific to BD, which has been suggested in previous studies of youth with and at-risk for BD [45, 46]. While preliminary, our findings highlight potential targets and risk factors that could be used to distinguish youth at risk for BD from youth at risk for MDD, and also possibly improve approaches to prevention and treatment selection for youth at familial risk for mood disorders.

**Supplementary Table 1.** Task and Motion Performance

| **Monetary Incentive Delay (MID) Task Performance** | | | | | |
| --- | --- | --- | --- | --- | --- |
|  | BD-risk (n=40) | MDD-risk (n=41) | HC (n=45) | *F* | *p* |
| Reaction Time for Rewards | 212.62 (35.81) | 205.29 (42.43) | 204.54 (27.01) | .59 | .56 |
| Reaction Time for Losses | 211.83 (38.14) | 209.52 (35.58) | 212.39 (27.44) | .08 | .92 |
| Reaction Time for Control | 215.61 (30.48) | 208.56 (37.83) | 218.20 (33.09) | .86 | .42 |
| Accuracy for Rewards (percent correct) | .59 (.23) | .62 (.19) | .62 (.19) | .25 | .70 |
| Accuracy for Losses (percent correct) | .56 (.23) | .59 (.16) | .61 (.21) | .73 | .49 |
| Accuracy for Control (percent correct) | .55 (.21) | .61 (.17) | .60 (.19) | 1.03 | .36 |
| Total Earned | 8.26 (23.81) | 13.04 (18.10) | 13.67 (21.32) | .79 | .46 |
| Motion: Absolute mean displacement (mm) (mean, SD) | .37 (.31) | .29 (.19) | .35 (.28) | .92 | .40 |

*Note.* Values indicate the Mean (SD) unless otherwise noted. BD-risk = youth at risk for bipolar disorder; F/U = follow-up; HC = healthy control youth; MDD-risk = youth at risk for a depressive disorder; mm = millimeter. Statistic computed using ANOVA.

**Supplementary Table 2. Participant SDQ Difficulties Characteristics**

| **Strengths and Difficulties Questionnaire** | | | | | | | | | | |
| --- | --- | --- | --- | --- | --- | --- | --- | --- | --- | --- |
|  | **BD-risk (n=40)** | | **MDD-risk (n=41)** | | **HC (n=45)** | | *F* | | *p* | |
|  | **Baseline** | **F/U** | **Baseline** | **F/U** | **Baseline** | **F/U** | **Baseline** | **F/U** | **Baseline** | **F/U** |
| Emotional problems scale | 2.00 (1.78) | 2.00 (2.42) | 2.29 (2.40) | 1.96 (2.07) | 1.84 (1.61) | .94 (1.24) | .29 | 2.87 | .75 | .06 |
| Peer problems scale | 1.00 (1.08) | .85 (1.06) | 1.19 (1.06) | 1.75 (1.78) | 1.00 (1.00) | .91 (.89) | .26 | 4.08 | .78 | **.02** |
| Conduct problems scale | 1.23 (1.48) | .70 (.95) | 1.13 (1.54) | .83 (1.09) | 1.05 (1.31) | .33 (.65) | .06 | 2.48 | .95 | .09 |
| Hyperactivity | 3.15 (2.64) | 2.93 (1.80) | 4.00 (2.78) | 3.13 (2.13) | 1.95 (2.68) | 2.79 (1.78) | 3.39 | .22 | **.04** | .80 |

*Note.* Values indicate the Mean (SD) unless otherwise noted. Statistic computed using ANOVA. F/U = follow-up.

**Supplementary Table 3.** Feature Sets

| Feature Set Number | Unique Neural Features | Constant Features |
| --- | --- | --- |
| 1 | Thalamus Activation  Thalamus- Left VLPFC Functional Connectivity  Thalamus-Left Angular Gyrus Functional Connectivity  Thalamus- Left Middle Frontal Gyrus Functional Connectivity  Thalamus- Left Superior Temporal Gyrus Functional Connectivity | Ethnicity  Gender  Age  CDRS  YMRS  Impulsivity  Approach-Withdrawal |
| 2 | Left Cerebellum Activation  Right Cerebellar Crus II Activation |

*Note.* CDRS = Children’s Depression Rating Scale; F/U = follow-up; VLPFC = ventrolateral prefrontal cortex; YMRS = Young Mania Rating Scale.

**
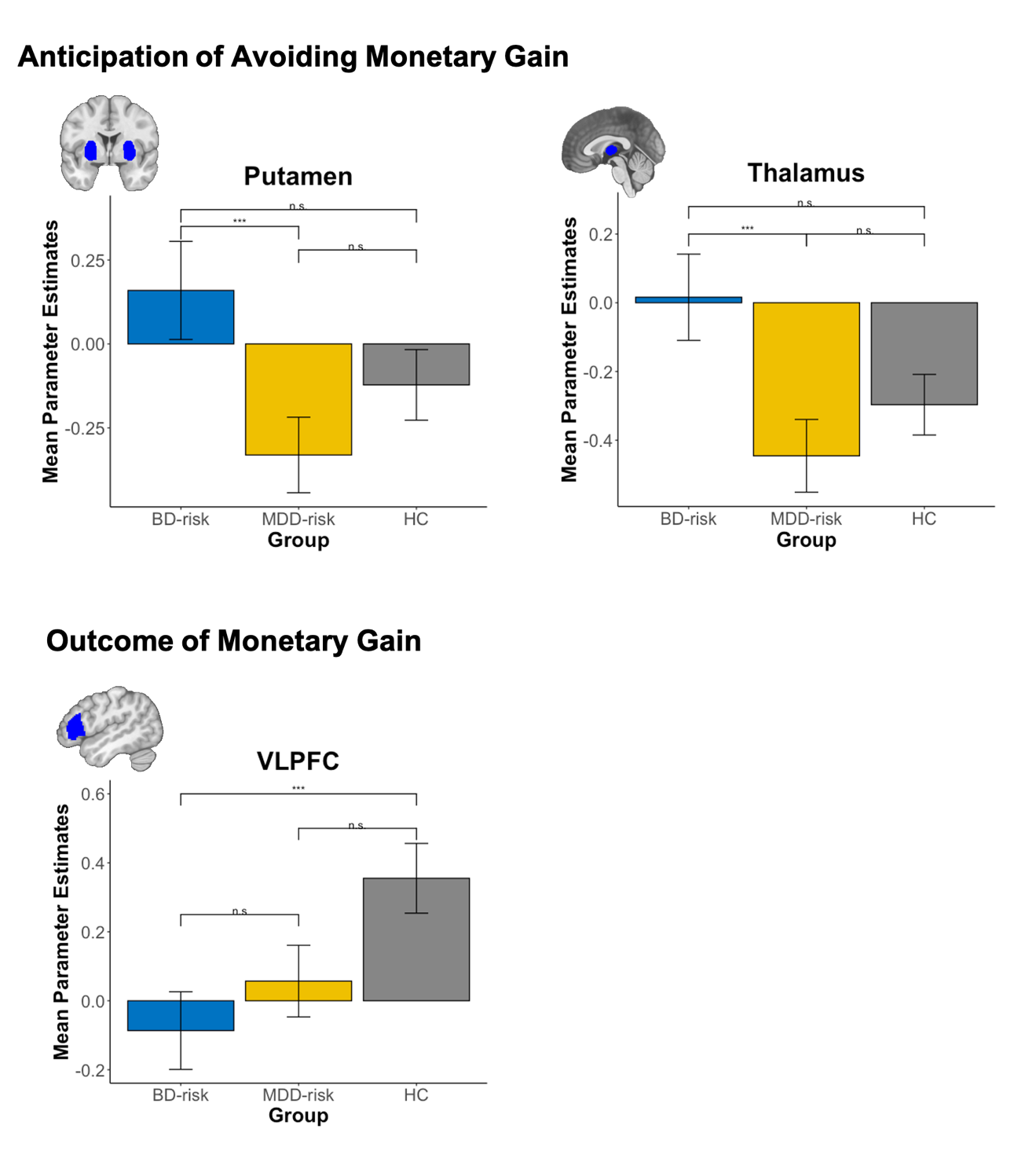
**

**Supplementary Figure 1. Group Differences Within Region of Interests During Reward Processing.** BD-risk had increased activation in the putamen and thalamus compared to MDD-risk and HC during anticipation of monetary avoiding monetary loss > anticipation of no monetary loss. In addition, BD-risk had reduced activation in the VLPFC compared to MDD-risk and HC during outcome of monetary gain versus no monetary gain or loss. Legend: blue: BD-risk, yellow: MDD-risk; gray: HC. Left side of image corresponds to the left hemisphere. Error bars are standard errors of the mean. n.s. = not significant; * *p* < .05, ** *p* < .01, *** *p* < .001.


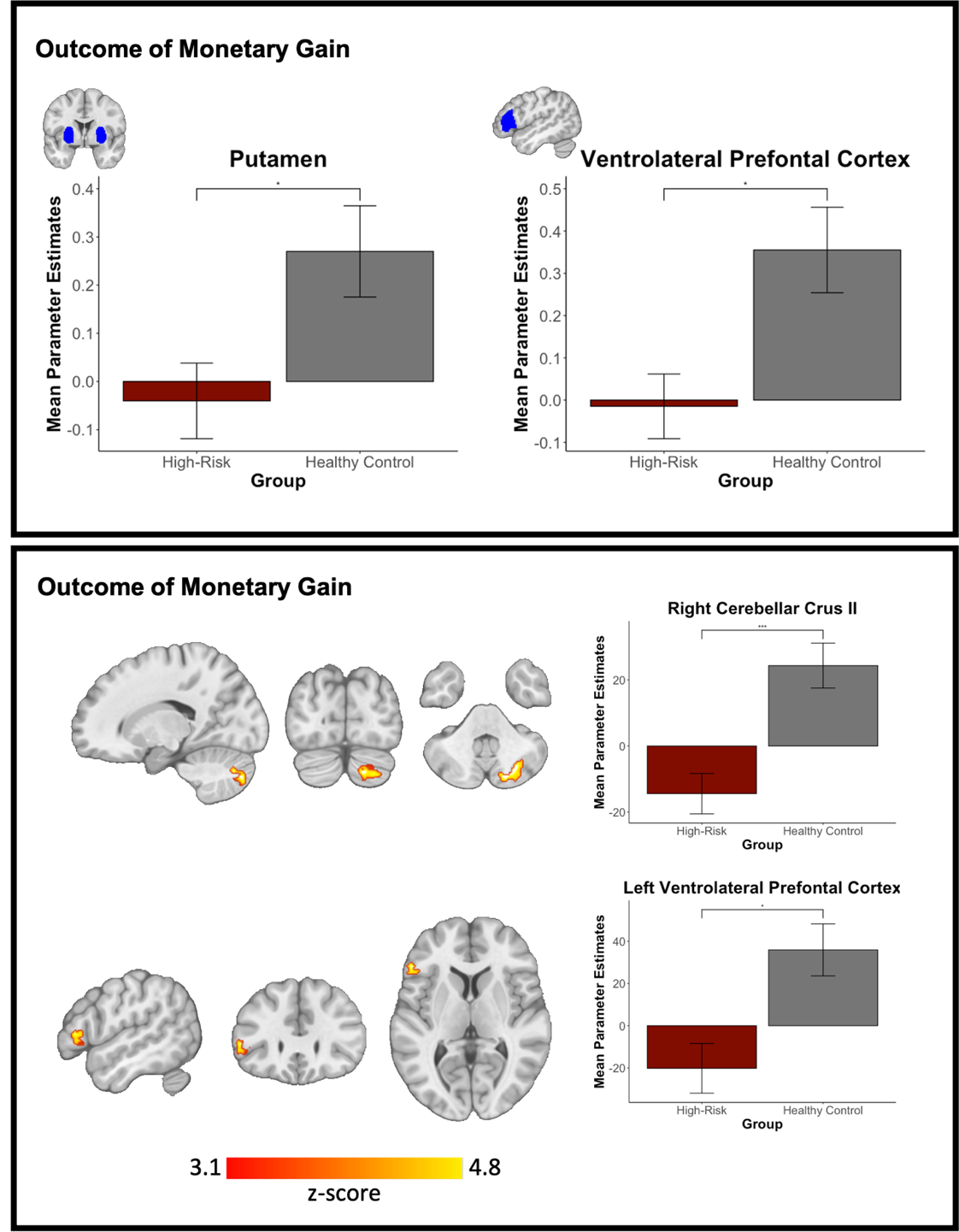


**Supplementary Figure 2. Significant High-Risk Group Differences Within Region of Interests and Whole-brain Clusters During Reward Processing.** High-Risk had decreased activation in the putamen and ventrolateral prefrontal cortex compared to Healthy Control during outcome of monetary gain > outcome of no monetary gain. Whole-brain, High-Risk had reduced activation in the right cerebellar crus II and the left ventrolateral prefrontal cortex compared to the Healthy Control group during outcome of monetary gain > outcome of no monetary gain. *Z-* statistics images were thresholded (Z > 3.1) using corrected cluster significance threshold of *p* < .05. Legend: maroon: High-Risk, gray: Healthy Control. Left side of image corresponds to the left hemisphere. Error bars are standard errors of the mean. * *p* < .05, *** *p* < .001.


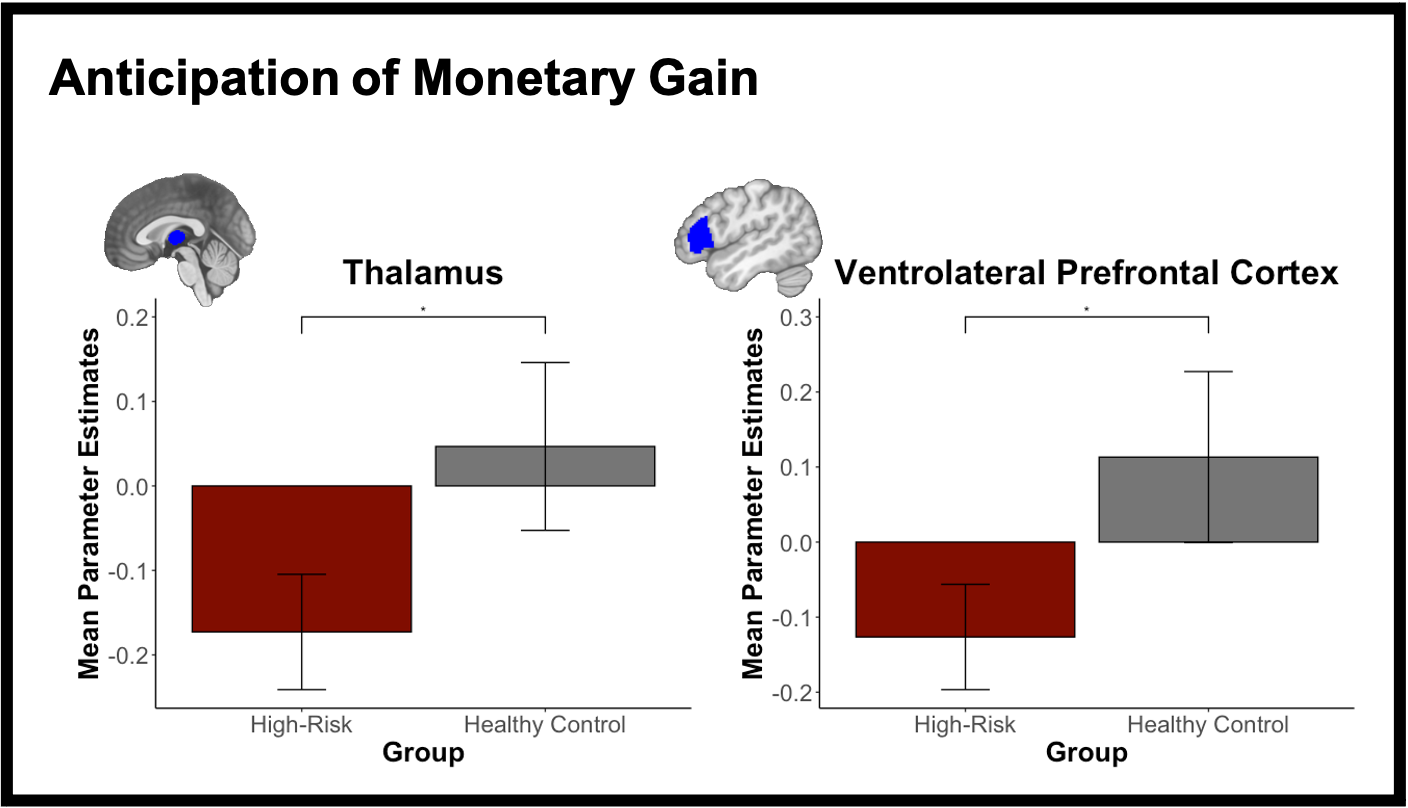


**Supplementary Figure 3. High-Risk Group Differences Within Region of Interests During Reward Processing.** High-Risk had decreased activation in the thalamus and ventrolateral prefrontal cortex compared to Healthy Control during anticipation of monetary gain > anticipation of no monetary gain. Legend: maroon: High-Risk, gray: Healthy Control. Error bars are standard errors of the mean. * *p* < .05.


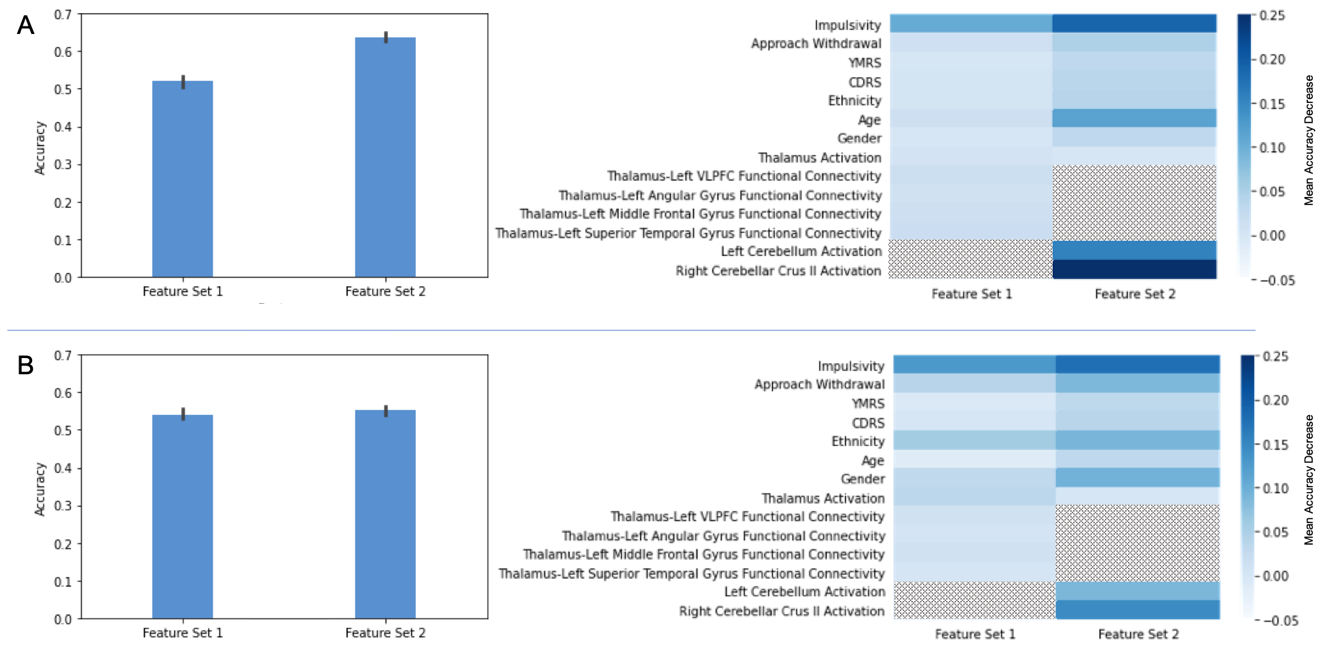


**Supplementary Figure 4. Feature Set Accuracy and Permutation Feature Importance** for (A) Random Forest and (B) Support Vector Machine. Features not included in the feature set are marked by grey stipple in the feature importance heat maps. CDRS = Children’s Depression Rating Scale; F/U = follow-up; VLPFC = ventrolateral prefrontal cortex; YMRS = Young Mania Rating Scale.

**References**

1. Weschler D. Weschler abbreviated scale of intelligence (WASI). Psychol Corp London. 1999. 1999.

2. Geller B, Zimerman B, Williams M, Bolhofner K, Craney JL, DelBello MP, et al. Reliability of the Washington University in St. Louis kiddie schedule for affective disorders and Schizophrenia (WASH-U-KSADS) mania and rapid cycling sections. J Am Acad Child Adolesc Psychiatry. 2001;40:450–455.

3. Kaufman J, Birmaher B, Brent D, Rao U, Flynn C, Moreci P, et al. Schedule for affective disorders and schizophrenia for school-age children-present and lifetime version (K-SADS-PL): Initial reliability and validity data. J Am Acad Child Adolesc Psychiatry. 1997;36:980–988.

4. Williams JBW, Gibbon M, First MB, Spitzer RL, Davies M, Borus J, et al. The Structured Clinical Interview for DSM-III-R (SCID). Arch Gen Psychiatry. 1992;49:630.

5. Goodman R. The Strengths and Difficulties Questionnaire: A Research Note. J Child Psychol Psychiatry. 1997;38:581–586.

6. Glover GH, Law CS. Spiral-in/out BOLD fMRI for increased SNR and reduced susceptibility artifacts. Magn Reson Med. 2001;46:515–522.

7. Jenkinson M, Beckmann CF, Behrens TEJ, Woolrich MW, Smith SM. FSL. Neuroimage. 2012;62:782–790.

8. Smith SM, Jenkinson M, Woolrich MW, Beckmann CF, Behrens TEJ, Johansen-Berg H, et al. Advances in functional and structural MR image analysis and implementation as FSL. Neuroimage. 2004;23:S208–S219.

9. Silverman MH, Jedd K, Luciana M. Neural networks involved in adolescent reward processing: An activation likelihood estimation meta-analysis of functional neuroimaging studies. Neuroimage. 2015;122:427–439.

10. Woolrich MW, Behrens TEJ, Beckmann CF, Jenkinson M, Smith SM. Multilevel linear modelling for FMRI group analysis using Bayesian inference. Neuroimage. 2004;21:1732–1747.

11. Worsley KJ. Statistical analysis of activation images. Ch 14, in Functional MRI: An Introduction to Methods, eds. P. Jezzard, P.M. Matthews and S.M. Smith. OUP, 2001; 2012.

12. Pedregosa, F. and Varoquaux G, Gramfort, A. and Michel V, Thirion, B. and Grisel O, Blondel, M. and Prettenhofer P, Weiss, R. and Dubourg V, Vanderplas, J. and Passos A, et al. Scikit-learn: Machine Learning in Python. J Mach Learn Res. 2011;12:2825–2830.

13. Abraham A, Pedregosa F, Eickenberg M, Gervais P, Mueller A, Kossaifi J, et al. Machine learning for neuroimaging with scikit-learn. Front Neuroinform. 2014;8:14.

14. Virtanen P, Gommers R, Oliphant TE, Haberland M, Reddy T, Cournapeau D, et al. SciPy 1.0: fundamental algorithms for scientific computing in Python. Nat Methods. 2020;17:261–272.

15. Crammer K, Singer Y. On the Algorithmic Implementation of Multiclass Kernel-based Vector Machines. vol. 2. 2001.

16. Vieira S, Garcia-Dias R, Lopez Pinaya WH. A step-by-step tutorial on how to build a machine learning model. Mach. Learn. Methods Appl. to Brain Disord., Elsevier; 2019. p. 343–370.

17. Breiman L. Random forests. Mach Learn. 2001;45:5–32.

18. Harris CR, Millman KJ, van der Walt SJ, Gommers R, Virtanen P, Cournapeau D, et al. Array programming with NumPy. Nature. 2020;585:357–362.

19. Miller CH, Sacchet MD, Gotlib IH. Support Vector Machines and Affective Science. Emot Rev. 2020;12:297–308.

20. Li X, Wong W, Lamoureux EL, Wong TY. Are linear regression techniques appropriate for analysis when the dependent (outcome) variable is not normally distributed? Invest Ophthalmol Vis Sci. 2012;53:3082–3083.

21. Balleine BW, Delgado MR, Hikosaka O. The role of the dorsal striatum in reward and decision-making. J Neurosci. 2007;27:8161–8165.

22. Haruno M, Kawato M. Different neural correlates of reward expectation and reward expectation error in the putamen and caudate nucleus during stimulus-action-reward association learning. J Neurophysiol. 2006;95:948–959.

23. Johnson SL. Mania and dysregulation in goal pursuit: a review. Clin Psychol Rev. 2005;25:241–262.

24. Fischer AS, Ellwood-Lowe ME, Colich NL, Cichocki A, Ho TC, Gotlib IH. Reward-circuit biomarkers of risk and resilience in adolescent depression. J Affect Disord. 2019;246:902–909.

25. Colich NL, Ho TC, Ellwood-Lowe ME, Foland-Ross LC, Sacchet MD, LeMoult JL, et al. Like mother like daughter: Putamen activation as amechanism underlying intergenerational risk for depression. Soc Cogn Affect Neurosci. 2017;12:1480–1489.

26. Gabbay V, Ely BA, Li Q, Bangaru SD, Panzer AM, Alonso CM, et al. Striatum-based circuitry of adolescent depression and anhedonia. J Am Acad Child Adolesc Psychiatry. 2013;52:628-641.e13.

27. Pizzagalli DA, Iosifescu D, Hallett LA, Ratner KG, Fava M. Reduced hedonic capacity in major depressive disorder: Evidence from a probabilistic reward task. J Psychiatr Res. 2008;43:76–87.

28. Sherman SM, Guillery RW. Exploring the thalamus and its role in cortical function, 2nd ed. Cambridge, MA, US: MIT Press; 2006.

29. Singh MK, Nimarko AF, Garrett AS, Gorelik AJ, Roybal DJ, Walshaw PD, et al. Changes in Intrinsic Brain Connectivity in Family-Focused Therapy Versus Standard Psychoeducation Among Youths at High Risk for Bipolar Disorder. J Am Acad Child Adolesc Psychiatry. 2020. 2020. https://doi.org/10.1016/j.jaac.2020.07.892.

30. Ladouceur CD, Diwadkar VA, White R, Bass J, Birmaher B, Axelson DA, et al. Fronto-limbic function in unaffected offspring at familial risk for bipolar disorder during an emotional working memory paradigm. Dev Cogn Neurosci. 2013;5:185–196.

31. Roberts G, Lord A, Frankland A, Wright A, Lau P, Levy F, et al. Functional Dysconnection of the Inferior Frontal Gyrus in Young People With Bipolar Disorder or at Genetic High Risk. Biol Psychiatry. 2017;81:718–727.

32. Jeganathan J, Perry A, Bassett DS, Roberts G, Mitchell PB, Breakspear M. Fronto-limbic dysconnectivity leads to impaired brain network controllability in young people with bipolar disorder and those at high genetic risk. NeuroImage Clin. 2018;19:71–81.

33. Singh MK, Kelley RG, Howe ME, Reiss AL, Gotlib IH, Chang KD. Reward processing in healthy offspring of parents with bipolar disorder. JAMA Psychiatry. 2014;71:1148–1156.

34. Manelis A, Ladouceur CD, Graur S, Monk K, Bonar LK, Hickey MB, et al. Altered functioning of reward circuitry in youth offspring of parents with bipolar disorder. Psychol Med. 2016;46:197–208.

35. Cho C, Smith D V., Delgado MR. Reward sensitivity enhances ventrolateral prefrontal cortex activation during free choice. Front Neurosci. 2016;10.

36. Chase HW, Phillips ML. Elucidating Neural Network Functional Connectivity Abnormalities in Bipolar Disorder: Toward a Harmonized Methodological Approach. Biol Psychiatry Cogn Neurosci Neuroimaging. 2016;1:288–298.

37. Whitton AE, Treadway MT, Pizzagalli DA. Reward processing dysfunction in major depression, bipolar disorder and schizophrenia. Curr Opin Psychiatry. 2015;28:7–12.

38. Goldstein BI, Birmaher B, Carlson GA, DelBello MP, Findling RL, Fristad M, et al. The International Society for Bipolar Disorders Task Force report on pediatric bipolar disorder: Knowledge to date and directions for future research. Bipolar Disord. 2017;19:524–543.

39. Findling RL, Stepanova E, Youngstrom EA, Young AS. Progress in diagnosis and treatment of bipolar disorder among children and adolescents: An international perspective. Evid Based Ment Health. 2018;21:177–181.

40. Axelson D, Birmaher B, Strober M, Gill MK, Valeri S, Chiappetta L, et al. Phenomenology of children and adolescents with bipolar spectrum disorders. Arch Gen Psychiatry. 2006;63:1139–1148.

41. Sandstrom A, MacKenzie L, Pizzo A, Fine A, Rempel S, Howard C, et al. Observed psychopathology in offspring of parents with major depressive disorder, bipolar disorder and schizophrenia. Psychol Med. 2020;50:1050–1056.

42. Platt B, Waters AM, Schulte-Koerne G, Engelmann L, Salemink E. A review of cognitive biases in youth depression: attention, interpretation and memory. Cogn Emot. 2017;31.

43. Gao S, Calhoun VD, Sui J. Machine learning in major depression: From classification to treatment outcome prediction. CNS Neurosci Ther. 2018;24:1037–1052.

44. Aydin O, Unal Aydin P, Arslan A. Development of Neuroimaging-Based Biomarkers in Psychiatry. Adv. Exp. Med. Biol., vol. 1192, Springer New York LLC; 2019. p. 159–195.

45. Singh MK, Chang KD, Kelley RG, Cui X, Sherdell L, Howe ME, et al. Reward processing in adolescents with bipolar I disorder. J Am Acad Child Adolesc Psychiatry. 2013;52:68–83.

46. Lee MS, Anumagalla P, Talluri P, Pavuluri MN. Meta-analyses of developing brain function in high-risk and emerged bipolar disorder. Front Psychiatry. 2014;5.
